# Regulation of Metastasis by microRNAs in Ovarian Cancer

**DOI:** 10.3389/fonc.2014.00143

**Published:** 2014-06-10

**Authors:** Yongchao Wang, Sangmi Kim, Il-man Kim

**Affiliations:** ^1^Vascular Biology Center, Medical College of Georgia, Georgia Regents University, Augusta, GA, USA; ^2^Cancer Center, Medical College of Georgia, Georgia Regents University, Augusta, GA, USA; ^3^Department of Biochemistry and Molecular Biology, Medical College of Georgia, Georgia Regents University, Augusta, GA, USA

**Keywords:** ovarian cancer, miRs, cancer stem cells, epithelial–mesenchymal transition, extracellular matrix, angiogenesis

## Abstract

Ovarian cancer (OC) is the second most common and the most fatal gynecologic cancer in the United States. Over the last decade, various targeted therapeutics have been introduced but there has been no corresponding improvement in patient survival mainly because of the lack of effective early detection methods. microRNAs (miRs) are small, non-coding RNAs that regulate gene expression post-transcriptionally. Accumulating data suggest central regulatory roles of miRs in modulating OC initiation, progression, and metastasis. More recently, aberrant miR expression has been also associated with cancer stem cell (CSC) phenotypes and development of CSC chemo-resistance. Here, we review recent advances on miRs and OC metastasis and discuss the concept that miRs are involved in both CSC transformation and subsequent OC metastasis. Finally, we describe the prevalence of circulating miRs and assess their potential utilities as biomarkers for OC diagnosis, prognosis, and therapeutics.

## Introduction

Ovarian cancer (OC) is the second most common gynecologic cancer and the deadliest malignancy among women in the United States (1). The current standard treatment rests on surgery followed by platinum-based chemotherapy. Early disease may be successfully removed with surgery alone, but most patients with OC are diagnosed at later stages. Advanced diseases, especially those metastasized, require complex treatment and management, accounting for high morbidity and mortality associated with OC. Cancer antigen 125 (CA-125), the currently available OC diagnostic marker, can only be adopted in advanced stages and has limited utility due to its lack of sensitivity and specificity (2–6). Therefore, development of novel biomarkers for early detection of OC is imperative and, toward the goal, research efforts to understand the detailed mechanism underlying its tumorigenesis and metastasis are warranted.

microRNAs (miRNAs or miRs) are small non-coding RNAs. They are first transcribed as primary miR transcripts (pri-miRs) and cropped by the Drosha complex into premature miRs (pre-miRs). Following the cleavage, exportin-5 mediates the nuclear translocation of the pre-miRs, which are further processed by the Dicer complex into mature miRs. The mature miRs are incorporated into RNA-induced silencing complex to execute gene-silencing effect either by repressing the translation or directly inhibiting messenger RNA (mRNA) stability. Notably, the 3′ untranslated regions (UTRs) of each mRNA usually have multiple miR binding sites and conversely each miR has multiple or even up to hundreds of target mRNAs as well. Therefore, even the subtle change of miR expression levels could be amplified in effect and consequently alters the functionality and phenotype of targeted cells by adjusting their repertoires. The abnormal expression of miRs in cancer leads to significant pathological consequences. Oncogenesis is conferred either by overexpression of miRs targeting tumor suppressors or loss of miRs that act as tumor suppressors. Recently, the involvement of miRs in tumorigenesis and metastasis of OC has been increasingly reported and potential utility of miRs in early detection of OC has been proposed (7–15).

## microRNAs and Ovarian Cancer Stem Cells

**Figure 1:**
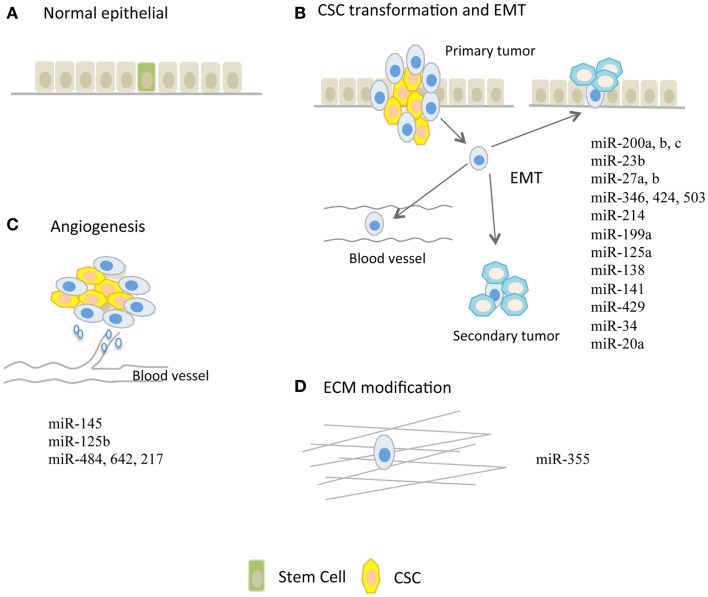
**microRNAs regulating tumor metastasis in ovarian cancer**. **(A)** Normal epithelium and potentially harbored stem cells. **(B)** miRs that are implicated in modulation of cancer stem cell (CSC) transformation and epithelial–mesenchymal transition (EMT) process. **(C)** miRs that are involved in tumor angiogenesis. Tumor angiogenesis, which is required for further tumor growth, is proven to be a handy process that facilitates tumor metastasis. **(D)** miR-355 that increases cell–extracellular matrix (ECM) interaction.

A subpopulation of stem-like cells have been isolated and identified in several distinct malignancies. These stem-like cells, named cancer stem cells (CSCs), are characterized as possessing the unlimited self-renewal capacity and pluripotency and thus, they could differentiate into various cell types and confer the heterogeneity of tumor cells (16). It is speculated that CSCs are evolved from resident stem cells harbored in tissues and are suggested to be genetically dynamic during tumor progression (17). Alternatively, epithelial–mesenchymal transition (EMT) could render CSC formation as illustrated in Figures 1A,B.

Since Bapat et al. first reported the involvement of CSCs in ovarian tumorigenesis (18), CSCs have been increasingly documented in OC cell lines, and tumor tissues and serum of patients (7–12). Using various markers characterizing ovarian CSCs such as CD44, 117, 133, 24, and aldehyde dehydrogenase-1 A1 (ALDH1), several studies suggest that the central regulatory roles of miRs in OC tumorigenesis are derived from their involvement in genetic alteration of CSCs that affects the functionality of CSCs, as summarized in Table 1.

**Table 1:** **microRNAs aberrantly expressed in ovarian cancer and their target genes**.

miRNA	Targets	Effects on tumor metastasis	Reference
**CSC-RELATED miRs**			
miR-23b	N/I	Enhances CSC transformation	(9)
miR-27a, b	VEGF, Cox2, and Sp1	Enhance CSC transformation and angiogenesis	(9, 19)
miR-214	P53/Nanog	Enhances CSC transformation	(10)
miR-346	N/I	Enhances CSC transformation	(9)
miR-424	N/I	Enhances CSC transformation	(9)
miR-503	N/I	Enhances CSC transformation	(9)
miR-199a	CD44, mTOR, HIF-1α, VEGF, HER2, and HER3	Inhibits CSC transformation and angiogenesis	(11, 12, 20)
miR-200a	ZEB-2	Inhibits CSC transformation and EMT process	(8, 15)
miR-200c	ZEB-1 and vimentin	Inhibits CSC transformation and EMT process	(7, 15)
**EMT-RELATED miRs**			
miR-20a	PTEN	Enhances EMT process	(21)
miR-34	N/I	Inhibits EMT process	(22, 23)
miR-125a	AT-rich interactive domain 3B (ARID3B)	Inhibits EMT process	(13)
miR-138	SRY-related high-mobility group box (SOX4) and HIF-1α	Inhibits EMT process	(14)
miR-141	N/I	Inhibits EMT process	(15)
miR-200b	N/I	Inhibits EMT process	(15)
miR-429	N/I	Inhibits EMT process	(15)
**ECM-RELATED miR**			
miR-355	Tenascin C (TNC)	Increases cell–ECM interaction	(24)
**ANGIOGENESIS-RELATED miRs**			
miR-22	N/I	Increases angiogenesis	(25)
miR-150, 146a	N/I	Increase angiogenesis	(26)
miR-182	BRCA1	Increases angiogenesis	(27)
miR-124	N/I	Decreases angiogenesis	(28)
miR-125b	HIF-1α, VEGF, HER2, and HER3	Decreases angiogenesis	(20)
miR-145	p70S6K1, HIF-1α, and VEGF	Decreases angiogenesis	(29)
miR-484, 642, 217	VEGFB and VEGFR2	N/I	(30)

*N/I, not identified*.

Chen et al. compared the expression levels of miR-200c between CD117^+^CD44^+^ ovarian CSCs and CD117^−^CD44^−^ CSCs and found that miR-200c was present predominantly in CD117^−^CD44^−^ CSCs. Overexpression of miR-200c in CD117^+^CD44^+^ CSCs significantly down-regulated the expression of ZEB-1 and vimentin, leading to an up-regulation of E-cadherin expression and dramatic reduction in the capacity of colony formation, migration, and invasion *in vitro*. The miR-200c overexpression also decreased the magnitude of metastasis in xenograft models of CD117^+^CD44^+^ CSCs (7). Similarly, miR-200a was also down-regulated in ovarian CD133^+^ CSCs than in CD133^−^ CSCs, and gain-of-function of miR-200a in CD133^+^ cells inhibited the migration and invasion of CD133^+^ ovarian CSCs. Mechanistically, ZEB-2 was shown to be the target of miR-200a (8). Xu et al. examined the role of miR-214 on formation of ovarian CSC phenotype, and found that enforced expression of miR-214 targeted P53/Nanog axis and contributed to ovarian CSC confluence and the ability of self-renewal (10).

microRNAs may also underlie the mechanism of CSC-induced development of chemo-resistance. Park et al. showed overexpression of miR-23b, miR-27a, miR-27b, miR-346, miR-424, and miR-503 in ALDH1^+^ and chemoresistant OC cells, and among those, miR-27 expression level was also correlated with metastasis magnitude of OC (9). In human OC cells, miR-199a repressed CD44 expression and inhibited the proliferation, migration, and invasion of CD44^+^CD117^+^ ovarian CSCs. The inhibition of CD44 by miR-199a reduced the expression of the multidrug resistance gene *ABCG2*, and thereby increased the chemosensitivity of ovarian CSCs (11). In addition, miR-199a was also implicated in cisplatin resistance by which its inhibition increased mTOR expression and decreased cisplatin-induced apoptosis *in vitro* (12).

## microRNAs and Epithelial–Mesenchymal Transition

Epithelial–mesenchymal transition in tumor is a process in which tumor cells loosen various interactions including cell–cell and cell–matrix, and are ready to detach, migrate, and metastasize through blood or lymph vessels (13, 21, 23). EMT is thought to be a crucial event that leads to tumor metastasis, possibly by increasing stem-like properties in cancer cells and facilitating their seeding in distance to form secondary tumors as illustrated in Figures 1A,B. Accumulating data points to regulatory roles of miRs on EMT phenotype, which is controlled by canonical pathways such as WNT and transforming growth factor pathways in OC as shown in Table 1.

microRNA-125a is widely reported as a tumor suppressor in various malignancies including OC. Karen et al. showed that miR-125a can inhibit EMT process in which overexpression of miR-125a induced the reversal of EMT in highly invasive OC cells (13). Using multiple biochemical approaches, epidermal growth factor receptor (EGFR) was proposed as a signaling pathway to suppress miR-125a expression via ETS family transcription factor, PEA3 in that study. Moreover, AT-rich interactive domain 3B (ARID3B) was shown to be the target of miR-125a to exert the EMT repression function (13). Similarly, miR-138 was down-regulated in highly invasive OC cell lines whereas *in vivo* overexpression of miR-138 inhibited OC metastasis (14). miR-138 has been shown to directly repress the expression of genes associated with EMT phenotype such as SRY-related high-mobility group box (SOX4) and hypoxia-inducible factor-1α (HIF-1α). According to this study, loss of HIF-α decreases slug expression through proteasome-mediated degradation, whereas EGFR is inhibited by knockdown of SOX4 (14).

In addition, miR-200 family including miR-200a, b, c, miR-141, and miR-429 are all dysregulated in OC cells undergoing EMT. For instance, the expression levels of miR-141 and miR-429 were significantly lower in mesenchymal-like and more metastatic HEY cells compared with epithelial-like and less invasive OVCAR3 cells. Forced overexpression of miR-429 and miR-141 in HEY cells was able to repress the signature characteristics of mesenchymal-like cells but activate those of epithelial-like cells (15). This finding indicates that miR-200 family not only inhibits CSC phenotypical formation but also acts as a gatekeeper to prevent OC cells from detaching and intravasation. Interestingly, cancer-associated miRs are often found in downstream of tumor suppressors or oncogenes. For example, miR-34, a tumor suppressor, is trans-activated by P53 (22). The expression of miR-34 is lower in OC compared with normal tissue and is further down-regulated with disease progression. Reconstitution of miR-34 results in decreased proliferation, progression/invasion, and reversal of EMT (23). Although most of miRs uncovered in ovarian EMT confer repressive effects on EMT (i.e., MET), miR-20a has been shown to down-regulate the expression of PTEN, a tumor suppressor and consequently render ovarian CSC properties and EMT phenotypical transformation (21).

## microRNAs and Extracellular Matrix Modification

Extracellular matrix (ECM) is composed of interlocking fibrous proteins and glycosaminoglycans (GAGs), which provide structural and biochemical support for cells surrounded as illustrated in Figure 1D. Tumors achieve invasion and metastasis through modulating the microenvironment, in particular the ECM that surrounds them (31–33). Aberrant expression of miRs could render tumor cells metastatic capacity through destruction of ECM as shown in Table 1. For example, in glioma, miR-21 was suggested to be an oncomiR because of its ability to repress the expression of RECK and TIMP3 which are the inhibitors of matrix metalloproteinase (MMPs), thereby conferring the metastatic capabilities (34). In human hepatocellular cancer (HCC), miR-21 also repressed PTEN expression and consequently increased focal adhesion kinase phosphorylation and expression of matrix MMP-2 and 9 (35). In addition to the regulation of canonical MMPs, some miRs are involved in cell–ECM interactions and expression of ECM components. The glycoprotein tenascin C (TNC), a protein that inhibits the interaction between cell–ECM, was shown to be a target of miR-355, and loss of miR-355 potentiated tumor cells’ metastatic abilities (31). miR-29c directly repressed the expression of ECM-related genes such as laminin gamma 1 and multiple collagens in nasopharyngeal carcinomas (NPCs) (32).

While the involvement of miRs in ECM formation and maintenance is increasingly reported in breast cancer, glioma, and HCC, the study in OC is still emerging. Interestingly, Sorrentino et al. demonstrated that miR-355, which has been shown to inhibit tumor metastasis by targeting TNC expression (31, 33, 36) was down-regulated in three OC cell lines resistant to paclitaxel and one cell line resistant to cisplatin, suggesting potential connection between ECM alteration and EMT transformation that can lead to chemo-resistance in OC (24).

## microRNAs and Angiogenesis

Tumor angiogenesis is the vital process through which tumors get further progressed and transformed from benign states to malignant states. It is contentious whether angiogenesis is necessary to tumor metastasis, but this handy process during tumor progression facilitates cells to reach and disseminate via blood circulation (19, 20, 29) as illustrated in Figure 1C. In response to hypoxic conditions, tumor cells during progression increase the expression of miRs that sustain tumor growth and neo-angiogenesis, or decrease the expression of miRs that repress neo-angiogenesis. For example, the up-regulated miR-27a is able to inhibit ZBTB10 expression and thereby indirectly regulates the expression levels of vascular endothelial growth factor (VEGF) and VEGF receptor (VEGFR). Conversely, miR-16, miR-15b, miR-20a, and miR-20b that repress angiogenesis through modulation of VEGF and VEGFR levels are consistently down-regulated in tumor cells. More recent studies shed light on the roles of miRs in OC angiogenesis as summarized in Table 1.

Xu et al. has shown that miR-145 was repressed in OC tissues and cell lines due to its inhibitory effect on neo-angiogenesis (29). A mechanistic study demonstrated that miR-145 was able to repress p70S6K1 expression and thereby inhibited the expression of both HIF-1α and VEGF. Similarly, miR-125b and miR-199a were shown to be tumor-suppressive miRs by targeting HIF-1α and VEGF and consequently reduced angiogenesis (20). In addition, HER2 and HER3 were suggested to be the direct targets of miR-125b and miR-199a because overexpression of miR-125b and miR-199a led to the failure of angiogenesis in xenograft models of OC, which is mediated by these two genes. Some miRs such as miR-217, miR-484, and miR-642 have been shown to directly modulate VEGFB and VEGFR2 pathways and predict tumor chemo-resistance (30). Ablation of miR-27a also repressed the expression of VEGF as well as Cox2 and Sp1. In particular, miR-27a appears to play a central role in follicle-stimulating hormone (FSH)-mediated angiogenesis in OC (19).

## Circulating microRNAs in Ovarian Cancer

Ovarian cancer is the most fatal gynecologic malignancy among women in the Unites States. Current treatment is based on surgery in combination with platinum-based chemotherapy. However, once patients with OC are diagnosed at advanced stages, there is limited availability of effective treatments. Therefore, the discovery and adoption of early detection biomarker is critical for the improved outcome. Cancer antigen 125 (CA-125) is the most commonly used marker to detect OC, monitor the response of OC to treatment, and predict patients’ prognosis after treatment. However, due to its lack of sensitivity, CA-125 is only detectable when patients are advanced to late stages. Furthermore, serum CA-125 levels are non-specifically elevated in patients with other cancers including cancers of lung, breast, and gastrointestinal tract (2–6).

Lawrie et al. first described that the serum levels of miR-21 were correlated with relapse-free survival in patients with diffuse large B-cell lymphoma (37). Subsequently, serum miR-141 levels have been used to monitor patients with prostate cancer. Using mouse prostate cancer xenograft models, the presence of tumor-derived miR-141 in mouse serum was confirmed (38). Chen et al. compared miR expression signature in patients with lung and colorectal cancer with healthy subjects and found that miR-25 and miR-223 were overexpressed in cancer patients (39, 40). Circulating miRs were also detected in patients with HCC, non-small-cell lung cancer (NSCLC), prostate cancer, breast cancer, and gastric cancer (41). Among patients with OC, miR-21, miR-29a, miR-92, miR-93, miR-99b, miR-126, miR-127, and miR-155 were aberrantly expressed in blood compared to healthy controls (40).

Because miRs were detected in patients’ serum even at early stage of various cancers, miR signatures of tumor-derived exosomes or other microvesicles have been proposed as diagnostic biomarkers of many cancers (42–44). Unlike those synthetic RNAs that are degraded by RNases in blood, miRs are incorporated into a membrane-enclosed complex and resistant to RNases in plasma. In addition, the levels of miRs in serum have been shown to be particularly stable against temperature and pH changes, rendering miRs as reliable diagnostic, prognostic, and predictive biomarkers (39, 45).

## Conclusion and Perspective

Metastasis is a multifactorial complex process in which tumor cells gain CSC-like properties and undergo EMT phenotypical changes through degrading ECM to disseminate into non-adjacent tissues with bloodstream. microRNAs play central roles in initiation, progression, invasion, and metastasis of cancer. While most miR signatures in cancers are involved in both tumor growth and metastasis, there are a few groups of them that were important for tumor metastasis, while exerting no effects on tumor proliferation (46). For example, miR-22 has just been linked to progression of OC to late stages, but no effects on cell viability and apoptosis (25). miR-150 and miR-146a are highly expressed in omental lesion compared to primary tumor sites and enhance spheroid formation to promote OC metastasis and chemoresistance to cisplatin (26). In addition, miR-124 is down-regulated in clinical OC specimens compared to adjacent normal tissues and correlates with the severity of metastasis both *in vivo* and *in vitro* (28). miR-182 is shown to be oncogenic, particularly in high-grade serous ovarian carcinoma (HG-SOC). miR-182 targets breast cancer 1 (BRCA1) and metastasis suppressor 1 (MTSS1) to impair the repair of DNA double-strand breaks, but simultaneously enhances the expression of high-mobility group AT-hook2 (HMGA2) (27). Taken together, it is well-appreciated that miRs are able to alter metastasis status by modulating the expression of genes involved in CSC transformation, EMT, angiogenesis, and ECM as shown in Table 1 and Figure 1. As we also reviewed the research on circulating miRs, miRs can be promising biomarkers for OC. Further studies are warranted to investigate their potential diagnostic and therapeutic utilities for patients with OC.

## Conflict of Interest Statement

The authors declare that the research was conducted in the absence of any commercial or financial relationships that could be construed as a potential conflict of interest.

## References

[1] Siegel R DeSantis C Virgo K Stein K Mariotto A Smith T Cancer treatment and survivorship statistics, 2012. CA Cancer J Clin (2012) 62(4):220–41 10.3322/caac.21149 22700443

[2] Nossov V Amneus M Su F Lang J Janco JM Reddy ST The early detection of ovarian cancer: from traditional methods to proteomics. Can we really do better than serum CA-125? Am J Obstet Gynecol (2008) 199(3):215–23 10.1016/j.ajog.2008.04.009 18468571

[3] Bast RC Jr Xu FJ Yu YH Barnhill S Zhang Z Mills GB CA 125: the past and the future. Int J Biol Markers (1998) 13(4):179–87 10228898 10.1177/172460089801300402

[4] Bagan P Berna P Assouad J Hupertan V Le Pimpec Barthes F Riquet M Value of cancer antigen 125 for diagnosis of pleural endometriosis in females with recurrent pneumothorax. Eur Respir J (2008) 31(1):140–2 10.1183/09031936.00094206 17804443

[5] Sarandakou A Protonotariou E Rizos D Tumor markers in biological fluids associated with pregnancy. Crit Rev Clin Lab Sci (2007) 44(2):151–78 10.1080/10408360601003143 17364691

[6] Asher V Hammond R Duncan TJ Pelvic mass associated with raised CA 125 for benign condition: a case report. World J Surg Oncol (2010) 8:28 10.1186/1477-7819-8-28 20398372 PMC2861664

[7] Chen D Zhang Y Wang J Chen J Yang C Cai K microRNA-200c overexpression inhibits tumorigenicity and metastasis of CD117+CD44+ ovarian cancer stem cells by regulating epithelial-mesenchymal transition. J Ovarian Res (2013) 6(1):50 10.1186/1757-2215-6-50 23842108 PMC3729499

[8] Wu Q Guo R Lin M Zhou B Wang Y microRNA-200a inhibits CD133/1+ ovarian cancer stem cells migration and invasion by targeting E-cadherin repressor ZEB2. Gynecol Oncol (2011) 122(1):149–54 10.1016/j.ygyno.2011.03.026 21529905

[9] Park YT Jeong JY Lee MJ Kim KI Kim TH Kwon YD microRNAs overexpressed in ovarian ALDH1-positive cells are associated with chemoresistance. J Ovarian Res (2013) 6(1):18 10.1186/1757-2215-6-18 23522567 PMC3637599

[10] Xu CX Xu M Tan L Yang H Permuth-Wey J Kruk PA microRNA miR-214 regulates ovarian cancer cell stemness by targeting p53/Nanog. J Biol Chem (2012) 287(42):34970–8 10.1074/jbc.M112.374611 22927443 PMC3471722

[11] Cheng W Liu T Wan X Gao Y Wang H microRNA-199a targets CD44 to suppress the tumorigenicity and multidrug resistance of ovarian cancer-initiating cells. FEBS J (2012) 279(11):2047–59 10.1111/j.1742-4658.2012.08589.x 22498306

[12] Wang Z Ting Z Li Y Chen G Lu Y Hao X microRNA-199a is able to reverse cisplatin resistance in human ovarian cancer cells through the inhibition of mammalian target of rapamycin. Oncol Lett (2013) 6(3):789–94 10.3892/ol.2013.1448 24137412 PMC3789061

[13] Cowden Dahl KD Dahl R Kruichak JN Hudson LG The epidermal growth factor receptor responsive miR-125a represses mesenchymal morphology in ovarian cancer cells. Neoplasia (2009) 11(11):1208–15 10.1593/neo.09942 19881956 PMC2767222

[14] Yeh YM Chuang CM Chao KC Wang LH microRNA-138 suppresses ovarian cancer cell invasion and metastasis by targeting SOX4 and HIF-1alpha. Int J Cancer (2013) 133(4):867–78 10.1002/ijc.28086 23389731

[15] Jabbari N Reavis AN McDonald JF Sequence variation among members of the miR-200 microRNA family is correlated with variation in the ability to induce hallmarks of mesenchymal-epithelial transition in ovarian cancer cells. J Ovarian Res (2014) 7(1):12 10.1186/1757-2215-7-12 24447705 PMC3901561

[16] Clevers H The cancer stem cell: premises, promises and challenges. Nat Med (2011) 17(3):313–9 10.1038/nm.2304 21386835

[17] Baccelli I Trumpp A The evolving concept of cancer and metastasis stem cells. J Cell Biol (2012) 198(3):281–93 10.1083/jcb.201202014 22869594 PMC3413352

[18] Bapat SA Mali AM Koppikar CB Kurrey NK Stem and progenitor-like cells contribute to the aggressive behavior of human epithelial ovarian cancer. Cancer Res (2005) 65(8):3025–9 10.1158/0008-5472.CAN-04-3931 15833827

[19] Lai Y Zhang X Zhang Z Shu Y Luo X Yang Y The microRNA-27a: ZBTB10-specificity protein pathway is involved in follicle stimulating hormone-induced VEGF, Cox2 and survivin expression in ovarian epithelial cancer cells. Int J Oncol (2013) 42(2):776–84 10.3892/ijo.2012.1743 23254909

[20] He J Jing Y Li W Qian X Xu Q Li FS Roles and mechanism of miR-199a and miR-125b in tumor angiogenesis. PLoS One (2013) 8(2):e56647 10.1371/journal.pone.0056647 23437196 PMC3577861

[21] Luo X Dong Z Chen Y Yang L Lai D Enrichment of ovarian cancer stem-like cells is associated with epithelial to mesenchymal transition through an miRNA-activated AKT pathway. Cell Prolif (2013) 46(4):436–46 10.1111/cpr.12038 23869765 PMC6496712

[22] Chang TC Wentzel EA Kent OA Ramachandran K Mullendore M Lee KH Transactivation of miR-34a by p53 broadly influences gene expression and promotes apoptosis. Mol Cell (2007) 26(5):745–52 10.1016/j.molcel.2007.05.010 17540599 PMC1939978

[23] Corney DC Hwang CI Matoso A Vogt M Flesken-Nikitin A Godwin AK Frequent downregulation of miR-34 family in human ovarian cancers. Clin Cancer Res (2010) 16(4):1119–28 10.1158/1078-0432.CCR-09-2642 20145172 PMC2822884

[24] Sorrentino A Liu CG Addario A Peschle C Scambia G Ferlini C Role of microRNAs in drug-resistant ovarian cancer cells. Gynecol Oncol (2008) 111(3):478–86 10.1016/j.ygyno.2008.08.017 18823650

[25] Li J Liang S Yu H Zhang J Ma D Lu X An inhibitory effect of miR-22 on cell migration and invasion in ovarian cancer. Gynecol Oncol (2010) 119(3):543–8 10.1016/j.ygyno.2010.08.034 20869762

[26] Vang S Wu HT Fischer A Miller DH MacLaughlan S Douglass E Identification of ovarian cancer metastatic miRNAs. PLoS One (2013) 8(3):e58226 10.1371/journal.pone.0058226 23554878 PMC3595263

[27] Liu Z Liu J Segura MF Shao C Lee P Gong Y miR-182 overexpression in tumourigenesis of high-grade serous ovarian carcinoma. J Pathol (2012) 228(2):204–15 10.1002/path.4000 22322863

[28] Zhang H Wang Q Zhao Q Di W miR-124 inhibits the migration and invasion of ovarian cancer cells by targeting SphK1. J Ovarian Res (2013) 6(1):84 10.1186/1757-2215-6-84 24279510 PMC3879084

[29] Xu Q Liu LZ Qian X Chen Q Jiang Y Li D miR-145 directly targets p70S6K1 in cancer cells to inhibit tumor growth and angiogenesis. Nucleic Acids Res (2012) 40(2):761–74 10.1093/nar/gkr730 21917858 PMC3258133

[30] Vecchione A Belletti B Lovat F Volinia S Chiappetta G Giglio S A microRNA signature defines chemoresistance in ovarian cancer through modulation of angiogenesis. Proc Natl Acad Sci U S A (2013) 110(24):9845–50 10.1073/pnas.1305472110 23697367 PMC3683704

[31] Orend G Chiquet-Ehrismann R Tenascin-C induced signaling in cancer. Cancer Lett (2006) 244(2):143–63 10.1016/j.canlet.2006.02.017 16632194

[32] Sengupta S den Boon JA Chen IH Newton MA Stanhope SA Cheng YJ microRNA 29c is down-regulated in nasopharyngeal carcinomas, up-regulating mRNAs encoding extracellular matrix proteins. Proc Natl Acad Sci U S A (2008) 105(15):5874–8 10.1073/pnas.0801130105 18390668 PMC2311339

[33] Negrini M Calin GA Breast cancer metastasis: a microRNA story. Breast Cancer Res (2008) 10(2):203 10.1186/bcr1867 18373886 PMC2397516

[34] Gabriely G Wurdinger T Kesari S Esau CC Burchard J Linsley PS microRNA 21 promotes glioma invasion by targeting matrix metalloproteinase regulators. Mol Cell Biol (2008) 28(17):5369–80 10.1128/MCB.00479-08 18591254 PMC2519720

[35] Meng F Henson R Wehbe-Janek H Ghoshal K Jacob ST Patel T microRNA-21 regulates expression of the PTEN tumor suppressor gene in human hepatocellular cancer. Gastroenterology (2007) 133(2):647–58 10.1053/j.gastro.2007.05.022 17681183 PMC4285346

[36] Tavazoie SF Alarcon C Oskarsson T Padua D Wang Q Bos PD Endogenous human microRNAs that suppress breast cancer metastasis. Nature (2008) 451(7175):147–52 10.1038/nature06487 18185580 PMC2782491

[37] Lawrie CH Gal S Dunlop HM Pushkaran B Liggins AP Pulford K Detection of elevated levels of tumour-associated microRNAs in serum of patients with diffuse large B-cell lymphoma. Br J Haematol (2008) 141(5):672–5 10.1111/j.1365-2141.2008.07077.x 18318758

[38] Mitchell PS Parkin RK Kroh EM Fritz BR Wyman SK Pogosova-Agadjanyan EL Circulating microRNAs as stable blood-based markers for cancer detection. Proc Natl Acad Sci U S A (2008) 105(30):10513–8 10.1073/pnas.0804549105 18663219 PMC2492472

[39] Chen X Ba Y Ma L Cai X Yin Y Wang K Characterization of microRNAs in serum: a novel class of biomarkers for diagnosis of cancer and other diseases. Cell Res (2008) 18(10):997–1006 10.1038/cr.2008.282 18766170

[40] Resnick KE Alder H Hagan JP Richardson DL Croce CM Cohn DE The detection of differentially expressed microRNAs from the serum of ovarian cancer patients using a novel real-time PCR platform. Gynecol Oncol (2009) 112(1):55–9 10.1016/j.ygyno.2008.08.036 18954897

[41] Hu Z Chen X Zhao Y Tian T Jin G Shu Y Serum microRNA signatures identified in a genome-wide serum microRNA expression profiling predict survival of non-small-cell lung cancer. J Clin Oncol (2010) 28(10):1721–6 10.1200/JCO.2009.24.9342 20194856

[42] Valadi H Ekstrom K Bossios A Sjostrand M Lee JJ Lotvall JO Exosome-mediated transfer of mRNAs and microRNAs is a novel mechanism of genetic exchange between cells. Nat Cell Biol (2007) 9(6):654–9 10.1038/ncb1596 17486113

[43] Rabinowits G Gercel-Taylor C Day JM Taylor DD Kloecker GH Exosomal microRNA: a diagnostic marker for lung cancer. Clin Lung Cancer (2009) 10(1):42–6 10.3816/CLC.2009.n.006 19289371

[44] Taylor DD Gercel-Taylor C microRNA signatures of tumor-derived exosomes as diagnostic biomarkers of ovarian cancer. Gynecol Oncol (2008) 110(1):13–21 10.1016/j.ygyno.2008.04.033 18589210

[45] Kroh EM Parkin RK Mitchell PS Tewari M Analysis of circulating microRNA biomarkers in plasma and serum using quantitative reverse transcription-PCR (qRT-PCR). Methods (2010) 50(4):298–301 10.1016/j.ymeth.2010.01.032 20146939 PMC4186708

[46] Ma L Teruya-Feldstein J Weinberg RA Tumour invasion and metastasis initiated by microRNA-10b in breast cancer. Nature (2007) 449(7163):682–8 10.1038/nature06174 17898713

